# Pharmacy students’ attitudes toward patient safety in Saudi Arabia: a cross-sectional study

**DOI:** 10.1186/s12909-020-02197-z

**Published:** 2020-08-18

**Authors:** Monira Alwhaibi, Yazed AlRuthia, Haya Almalag, Hadeel Alkofide, Bander Balkhi, Amani Almejel, Fahad Alshammari

**Affiliations:** 1 Department of Clinical Pharmacy, College of Pharmacy, King Saud University, Riyadh, 11149 Saudi Arabia; 2 Medication Safety Research Chair, College of Pharmacy, King Saud University, Riyadh, Saudi Arabia; 3 Pharmacoeconomics Research Unit, College of Pharmacy, King Saud University, Riyadh, Saudi Arabia

**Keywords:** Attitudes, Pharmacy students, Patient Safety

## Abstract

**Background:**

There is a growing recognition of the importance of teaching patient safety to medical students to improve healthcare and minimize patients’ harm; however, few studies evaluated the attitudes of pharmacy students toward patient safety. The purpose of this study was to explore the attitudes toward patient safety among pharmacy students in Saudi Arabia.

**Methods:**

A cross-sectional study was conducted among pharmacy students from four different universities using a self-administered questionnaire. The Attitudes to Patient Safety Questionnaire III (APSQ-III) was used to measure the attitude toward patient safety. The data were presented using descriptive statistics, such as percentages and means, and compared across gender using Student’s t-test.

**Results:**

All of the students who agreed to participate and signed the consent form have completed the questionnaire. Of the 347 pharmacy students who participated in the study; 63% were enrolled in the Doctor of Pharmacy Program and 37% were enrolled in the Bachelor of Pharmaceutical Sciences program. Only 46% of the participants received courses for patient safety mainly in the fourth year of their pharmacy program, and around 93% were interested to learn more about patient safety. A more positive attitude toward patient safety was reported in the domain of ‘confidence to report errors’, ‘working hours as error cause’, ‘patient involvement in reducing error’, and ‘team functioning’. However, most negative attitudes were reported in the domains of ‘Error inevitability’ and ‘Disclosure responsibility’. Gender differences were noticed in the attitude toward patient safety; female students had more positive attitudes in most domains of patient safety.

**Conclusions:**

Around one-half of the surveyed pharmacy students did not receive any courses on patient safety. Our findings emphasize the need for including patient safety courses in the curricula of the different pharmacy programs given the patient safety training importance in improving the quality of patient care.

## Background

Patient Safety is undoubtedly an important topic to teach students in the medical field. Medical students must be knowledgeable about the concept and applications of patient safety as they will have a future leading role in the culture of patient safety. The World Health Organization (WHO) has recognized this importance and has published a curriculum guide to integrating patient safety into the curriculum for teaching medical students [1]. The curriculum guide aims to support the worldwide implementation of patient safety education, promote the status of patient safety, and eventually prepare students for safe health practice. The guide covers several topics ranging from being an effective team player, infection control, medication safety, to system errors and engaging with patients and caregivers. Lucian Leape Institute has issued a report “Unmet Needs: Teaching Physicians to Provide Safe Patient Care”, which called for the development of patient safety evaluation systems for medical students to assess the milestones in patient safety [2].

Studies have found that receiving education on patient safety is associated with improved medical students’ patient safety knowledge, skills, and attitude [3–10]. In addition, systematic reviews of published articles have found that many medical schools have adopted a wide range of educational methods to promote patient and medication safety [11, 12]. For example, some universities provide patient safety courses using traditional lectures, simulation exercises, root cause analysis workshops, and other teaching methods [11, 12]. Several studies have reported a positive attitude toward patient safety among medical students [13–17]. Improving healthcare students’ patient safety knowledge, skills, and attitude can prepare future healthcare professionals to create and maintain a safe health care environment for patients, this can lead to decrease medication errors and improve patient safety [18, 19].

On the other hand, studies that explored pharmacy students’ attitudes toward patient safety are scarce. In a questionnaire-based cross-sectional study that was conducted among a group of pharmacy students in an Ethiopian university to explore their attitudes toward patients’ safety, the majority of students have expressed favorable attitudes toward patient safety. However, this study was conducted only in one university and among students in the fourth and fifth years of their pharmacy program [13]. Therefore, the findings of this study are not generalizable to other pharmacy students or pharmacy programs elsewhere, which necessitate the exploration of pharmacy students’ attitudes in other universities and parts of the world. Saudi Arabia has witnessed a fast transformation in both the structure and number of the pharmacy programs over the past decade. The bachelor in pharmaceutical sciences at King Saud University College of Pharmacy, which was the only program graduating pharmacists until 2007, is now being phased out and replaced by the Doctor of Pharmacy (PharmD) program with more patient care orientation. Additionally, the number of pharmacy colleges has increased from one college in 2002 to more than 27 colleges in 2018 [20]. However, no study has explored pharmacy students’ attitudes toward patient safety in Saudi Arabia or whether patient safety courses are offered consistently in all colleges of pharmacy in Saudi Arabia. The findings of this study can help in identifying pharmacy students’ attitudes towards patient safety which is essential for the design of the educational program which can untimely help in promoting the patient safety environment and safe practices among future healthcare professionals. Therefore, the aim of this study was to explore pharmacy students’ attitudes toward patient safety as well as whether patient safety courses are offered consistently in four different colleges of pharmacy in Saudi Arabia.

## Methods

### Study design and sample

A questionnaire-based cross-sectional study was conducted among pharmacy students in four different colleges of pharmacy in Saudi Arabia. The study sample comprised of pharmacy students who finished at least the first year of their pharmacy program (i.e. bachelor or doctor of pharmacy).

### Data collection

An advertisement to recruit pharmacy students was posted online at King Saud University Pharmacy Education Unit twitter account explaining the purpose of the study as well as informing interested participants about the venue, date and time where the questionnaire will be distributed and collected. Interested students in each of the four colleges have stopped by at the specified venue in their respective colleges and collected the questionnaire and were given 20 min to submit after signing the consent form that explained the purpose of the study and their right to withdraw from the study at any time. All of the students who agreed to participate and signed the consent form have completed the questionnaire. The permission to collect the data was granted by four different pharmacy colleges (ALmaarefa college of Science and Technology, King Saud University, Princess Nora University, Taibah University) in the cities of Riyadh and Almadina, Saudi Arabia. The study was approved by the institutional review board of Princess Nora University.

### Measures

#### Dependent variable

In our study, the dependent variable was “patient safety attitude” which was measured using the modified and validated version of the Attitudes to Patient Safety Questionnaire III (APSQ-III) [21]. This questionnaire has been used by several studies to assess attitudes to patient safety [13–17]. The survey consists of 26 items covering nine key patient safety factors: (1) patient safety training received; (2) error reporting confidence; (3) working hours as an error cause; (4) error inevitability; (5) professional incompetence as an error cause; (6) disclosure responsibility; (7) team functioning; (8) patient involvement in reducing error; and (9) the importance of patient safety in the curriculum. Responses to each item were rated on a five-point Likert-type scale ranging from 1 (strongly disagree) to 5 (strongly agree). A higher score indicated a more positive response to the patient safety factor. Items (11, 13–16, 24) were reverse scored, according to the instrument. Nine sub-scores were calculated for each participant corresponding to the nine patient safety factors.

#### Independent variables

Independent variables were the age in years, sex, type of pharmacy program, year of pharmacy education. Participants were also asked about previous education received about patient safety by asking, “Have you taken any courses for patient safety?” those who responded yes were asked, “What type of educational material have you received?” Participants were also asked “Are you interested in learning about patient safety?”

### Statistical analysis

Frequency and percentage were used to describe the data. Subgroup analysis was conducted to assess the attitude towards patient safety among female and male students using Student’s t-test. The significance level was set at α of 0.05. All statistical analyses were carried out using the Statistical Package for the Social Sciences (SPSS Inc., Chicago, IL, USA).

## Results

### Characteristics of the study sample

The number of participants in this study was 347 pharmacy students from four different universities (Table 1). The majority of the participants were between 22 to 25 years of age (*n* = 171; 49.3%), female (*n* = 219; 63.1%), and were enrolled in a PharmD program (*n* = 230; 66.3%).

**Table 1:** Characteristics of the Study Sample

	N	%
**Total**	347	100
**Age group**		
18–21 years	159	45.8
22–25 years	171	49.3
26–29 years	11	3.2
30 and older	3	0.9
**Gender**		
Female	219	63.1
Male	128	36.9
**Type of Pharmacy Program**		
Doctor of Pharmacy (Pharm. D.)	230	66.3
Bachelor of Pharmacy	112	32.3
**Region and University**		
AlMaarefa Colleges for Sciences and Technology	23	6.6
King Saud University	144	41.5
Princess Nora University	88	25.4
Taibah University	91	26.2
**Level /Grade of Pharmacy Program**		
Semester 1 OR 2	8	2.3
Semester 4	66	19.0
Semester 6	75	21.6
Semester 7 OR 8	99	28.5
Semester 10	49	14.1
Semester 12	48	13.8

Note: Based on 347 Pharmacy Students from four different universities

### Knowledge about patient Safety

Table 2 displays the current knowledge about patient safety. Only 46% of the participants received courses on patient safety. For students who received courses about patient safety, the majority covered this topic in the fourth year of pharmacy education. Most participants (*n* = 324; 93.4%) were interested to learn about patient safety in the pharmacy curriculum.

**Table 2:** Students’ Previous Exposure to Patient Safety

	N	%
**Total**	347	100
**Have you taken any courses for patient safety?**		
Yes	159	45.8
No	180	51.9
**If yes, in which year(s) did you take it?**		
1st year (preparatory)	20.0	5.8
2nd year	19.0	5.5
3rd year	31.0	8.8
4th year	62.0	17.9
5th year	20.0	5.8
6th year	1.0	0.3
**What type of educational material have you received?**		
Lectures	225	64.8
Simulation exercises	5	1.4
Workshops	6	1.7
Lectures, Workshops	16	4.6
Lectures, simulation exercises, root cause analysis	17	4.9
Lectures, Workshops, simulation exercises	13	3.8
Lectures, Workshops, simulation exercises, root cause analysis, other learning methods	7	2.1
**Are you interested in learning about patient safety?**		
Yes	324	93.4
No	20	5.8

Note: Based on 347 Pharmacy Students from four different universities

### Attitude towards patient Safety

Table 3 displays the attitude towards patient safety. In terms of patient training received, only one-half (55.3%) of the students agreed or strongly agreed that their training prepares them to understand the causes of medical errors. Regarding the reporting confidence, around 50% of pharmacy students are feeling comfortable reporting errors they had made and talk openly to their supervisors.

**Table 3:** Students’ Attitude Towards Patient Safety using the Modified Version of the Attitudes to Patient Safety Questionnaire III (APSQ-III)

Key Safety Factor Items	Strongly Disagree N (%)	Disagree N (%)	Undecided N (%)	Agree N (%)	Strongly Agree N (%)
**1. Patient safety training received**					
1. My training is preparing me to understand the causes of medical errors	27 (7.8)	46 (13.2)	45 (13.0)	98 (28.2)	94 (27.1)
2. I have a good understanding of patient safety issues as a result of my undergraduate medical training.	9 (2.6)	72 (20.7)	59 (17.0)	103 (29.7)	65 (18.7)
3. My training is preparing me to prevent medical errors	11 (3.2)	56 (16.1)	49 (14.1)	81 (23.3)	111 (32.0)
**2. Error reporting confidence**					
4. I would feel comfortable reporting any errors I had made, no matter how serious the outcome had been for the patient.	15 (4.3)	49 (14.1)	51 (14.7)	93 (26.8)	102 (29.4)
5. I would feel comfortable reporting any errors other people had made, no matter how serious the outcome had been for the patient.	11 (3.2)	38 (11.0)	73 (21.0)	97 (28.0)	92 (26.5)
6. I am confident I could talk openly to my supervisor about an error I had made if it had resulted in potential or actual harm to my patient	7 (2.0)	38 (11.0)	46 (13.3)	102 (29.4)	119 (34.3)
**3. Working hours as error cause**					
7. Shorter shifts for doctors will reduce medical errors	12 (3.5)	70 (20.2)	57 (16.4)	73 (21.0)	100 (28.8)
8. By not taking regular breaks during shifts doctors are at an increased risk of making errors	7 (2.0)	46 (13.3)	44 (12.7)	86 (24.8)	128 (36.9)
9. The number of hours doctors work increases the likelihood of making medical errors	7 (2.0)	44 (12.7)	58 (16.7)	78 (22.5)	123 (35.4)
**4. Error inevitability**					
10. Even the most experienced and competent doctors make errors.	8 (2.3)	42 (12.2)	42 (12.1)	81 (23.3)	138 (39.8)
11. A true professional does not make mistakes or errors	105 (30.3)	110 (31.7)	43 (12.4)	36 (10.4)	16 (4.6)
12. Human error is inevitable	41 (11.8)	79 (22.8)	73 (21.0)	54 (15.6)	60 (17.3)
**5. Professional incompetence as error cause**					
13. If people paid more attention at work, medical errors would be avoided	10 (2.9)	37 (10.7)	41 (11.8)	106 (30.5)	117 (3.7)
14. Most medical errors result from careless doctors	18 (5.2)	78 (22.5)	65 (18.7)	85 (24.5)	64 (18.4)
15. Medical errors are a sign of incompetence	12 (3.5)	65 (18.7)	91 (26.2)	84 (24.2)	55 (15.9)
**6. Disclosure responsibility**					
16. It is not necessary to report errors which do not result in adverse outcomes for the patient	97 (28.0)	81 (23.4)	51 (14.7)	42 (12.1)	37 (10.7)
17. Doctors have a responsibility to disclose errors to patients only if they result in patient harm	50 (14.4)	84 (24.6)	58 (16.7)	60 (17.3)	56 (16.1)
18. All medical errors should be reported	10 (2.9)	21 (8.9)	40 (11.5)	59 (17.0)	165 (47.6)
**7. Team functioning**					
19. Better multi-disciplinary teamwork will reduce medical errors	5 (1.4)	28 (8.4)	38 (11.0)	82 (23.6)	155 (44.7)
20. Teaching teamwork skills will reduce medical errors	5 (1.4)	26 (7.5)	40 (11.5)	71 (20.5)	166 (47.8)
**8. Patient involvement in reducing error**					
21. Patients have an important role in preventing medical errors	10 (2.9)	51 (14.7)	64 (18.4)	103 (29.7)	82 (23.6)
22. Encouraging patients to be more involved in their care can help to reduce the risk of medical errors occurring.	8 (2.3)	33 (9.5)	44 (12.7)	98 (28.2)	125 (36.0)
**9. Importance of patient safety in the curriculum**					
23. Teaching students about patient safety should be an important priority in medical students training	17 (4.9)	34 (9.8)	28 (8.1)	59 (17.0)	169 (48.7)
24. Patient safety issues cannot be taught and can only be learned by clinical experience when qualified	31 (8.9)	94 (27.1)	59 (17.0)	59 (17.0)	64 (18.4)
26. Learning about patient safety issues before I qualify will enable me to become a more effective doctor	18 (5.2)	35 (10.6)	31 (8.9)	69 (19.9)	154 (44.4)

Note: Based on 347 Pharmacy Students from four different universities

Two-third of pharmacy students disagreed or strongly disagreed that a true professional does not make errors, while one-third of the students agreed or strongly agreed that making more attention at work, errors can be avoided. In terms of disclosure responsibility, around 64% of the students agreed or strongly agreed that all medical errors should be reported.

Two-third of pharmacy students agreed or strongly agreed that teaching students about patient safety should be an important priority and learning about patient safety issues will enable them to become more effective.

### Gender differences in the attitude towards patient Safety

Table 4 displays the gender difference in the attitude towards patient safety. Female students had a more positive attitude in the domains of patient safety training received, error reporting confidence, and working hours as error cause, error inevitability, team functioning, and patient involvement in reducing error compared to their male counterparts. On the other hand, the male students had a more positive attitude in the domain of professional incompetence as error cause compared to their female counterparts. However, no significant difference in the attitude was observed among female students as compared to their male counterparts in the disclosure responsibility domain.

**Table 4:** Gender differences in Pharmacy Students’ Attitude Towards Patient Safety

Key Safety Factor Items	Mean	SD	Female	Male	***P***-value
			Mean ± SD	Mean ± SD	
**1. Patient safety training received**					
1. My training is preparing me to understand the causes of medical errors	4.44	1.581	4.7 (1.35)	3.9 (1.84)	< 0.001
2. I have a good understanding of patient safety issues as a result of my undergraduate medical training.	4.31	1.402	4.6 (1.27)	3.8 (1.50)	< 0.001
3. My training is preparing me to prevent medical errors	4.62	1.438	4.9 (1.26)	4.1 (1.63)	< 0.001
**2. Error reporting confidence**					
4. I would feel comfortable reporting any errors I had made, no matter how serious the outcome had been for the patient.	4.58	1.454	4.9 (1.63)	3.9 (1.54)	< 0.001
5. I would feel comfortable reporting any errors other people had made, no matter how serious the outcome had been for the patient.	4.62	1.312	4.9 (1.17)	4.2 (1.45)	< 0.001
6. I am confident I could talk openly to my supervisor about an error I had made if it had resulted in potential or actual harm to my patient	4.84	1.288	5.1 (1.08)	4.3 (1.49)	< 0.001
**3. Working hours as error cause**					
7. Shorter shifts for doctors will reduce medical errors	4.43	1.488	4.7 (1.42)	4.0 (1.53)	< 0.001
8. By not taking regular breaks during shifts doctors are at an increased risk of making errors	4.83	1.325	5.2 (1.10)	4.2 (1.50)	< 0.001
9. The number of hours doctors work increases the likelihood of making medical errors	4.78	1.321	5.1 (1.04)	4.1 (1.54)	< 0.001
**4. Error inevitability**					
10. Even the most experienced and competent doctors make errors.	4.87	1.363	5.3 (0.95)	4.1 (1.67)	< 0.001
11. A true professional does not make mistakes or errors	4.39	1.586	4.6 (1.53)	4.0 (1.64)	< 0.001
12. Human error is inevitable	3.79	1.651	3.8 (1.68)	3.7 (1.60)	0.437
**5. Professional incompetence as error cause**					
13. If people paid more attention at work, medical errors would be avoided	2.18	1.327	1.9 (1.11)	2.7 (1.55)	0.003
14. Most medical errors result from careless doctors	2.86	1.493	2.8 (1.46)	3.1 (1.55)	0.097
15. Medical errors are a sign of incompetence	2.80	1.350	2.6 (1.33)	3.1 (1.35)	0.003
**6. Disclosure responsibility**					
16. It is not necessary to report errors which do not result in adverse outcomes for the patient	4.00	1.798	4.0 (1.89)	3.9 (1.60)	0.689
17. Doctors have a responsibility to disclose errors to patients only if they result in patient harm	3.64	1.734	3.7 (1.80)	3.6 (1.61)	0.756
18. All medical errors should be reported	5.03	1.350	5.2 (1.21)	4.6 (1.52)	< 0.001
**7. Team functioning**					
19. Better multi-disciplinary teamwork will reduce medical errors	5.09	1.190	5.3 (0.95)	4.6 (1.43)	< 0.001
20. Teaching teamwork skills will reduce medical errors	5.13	1.217	5.5 (0.86)	4.5 (1.52)	< 0.001
**8. Patient involvement in reducing error**					
21. Patients have an important role in preventing medical errors	4.53	1.336	4.8 (1.05)	3.9 (1.59)	< 0.001
22. Encouraging patients to be more involved in their care can help to reduce the risk of medical errors occurring.	4.91	1.252	5.2 (0.92)	4.3 (1.56)	< 0.001
**9. Importance of patient safety in the curriculum**					
23. Teaching students about patient safety should be an important priority in medical students training	4.95	1.522	5.4 (1.11)	4.2 (1.85)	< 0.001
24. Patient safety issues cannot be taught and can only be learned by clinical experience when qualified	3.19	1.665	2.9 (1.64)	3.8 (1.56)	< 0.001
25. Learning about patient safety issues before I qualify will enable me to become a more effective doctor	4.89	1.495	5.3 (1.20)	4.2 (1.70)	< 0.001

Note: Based on 347 Pharmacy Students from four different universities

Mean ± SD: mean ± standard deviation

## Discussion

In general, pharmacy students showed positive attitudes toward patient safety, and a more positive attitude towards the patient safety was reported in the domain of ‘confidence to report errors’, ‘working hours as error cause’, ‘patient involvement in reducing error’, and the role of multi-disciplinary teamwork to reduce medical errors. However, negative attitudes were noticeable in the domains of ‘error inevitability’ and ‘disclosure responsibility’. These findings are consistent with the findings of other studies among medical and pharmacy students [13–17].

Importantly, the majority of the pharmacy students considered the courses about patient safety important in their training which was expressed in their positive attitude. In fact, a positive attitude toward patient safety has been associated with medical students’ motivation to participate in patient safety training [22]. This finding indicates that the pharmacy students are receptive to changes that would incorporate patient safety courses in their pharmacy programs’ curricula. The students’ recognition of the importance of the patient safety subject also served as a positive feedback to the stakeholders to integrate patient safety in the pharmacy curriculum in light of the national reform in higher education. Therefore, communicating the study findings to the stakeholders can positively impact the integration of the patient safety topic into the pharmacy students’ curriculum. In fact, patient safety research such as this study and studies among other healthcare students is a top propriety area of research to assess the extent and nature of the patient safety issue which is one of the top global priority research as reported by the WHO [23].

Further, this study found that only one-half of pharmacy students are feeling confident to report errors they had made or other people had made and talk openly to their supervisors. Therefore, it is important that pharmacy graduates have sufficient confidence to protect patients from potential harms or errors. Thus, pharmacy education has a critical role in the development of the knowledge skills, and confidence required of graduates to warrant they are well prepared to provide a safe health care environment for patients.

Furthermore, a noteworthy finding in this study was the gender difference in the attitude toward patient safety; female students had a more positive attitude in most of the patient safety domains. Males, however, scored higher in the domain of ‘Professional incompetence as error cause’. In the published literature, a study reported that female nursing students have higher patient safety competence in the classroom and clinical settings [24]. The limited information patient safety literature when it comes to gender differences warranty further exploration to reconcile the gap. Besides, the potential reasons for the gender difference in the attitude toward patient safety need to be explored in future studies. Also, it is important to study the impact of patient safety medical education on the attitude of female and male towards patient safety.

As patient safety has become a substantial issue for healthcare organizations that are determined to improve the quality of their healthcare services. Therefore, training pharmacy students about patient safety is needed to improve the quality of patient care. The present study results emphasize the importance of providing education and training on patient safety to pharmacists. Besides, the findings of the present study could serve as a reference for other universities planning to introduce patient safety education in their curriculum. National efforts in Saudi Arabia to incorporate patient safety in the medical curriculum has been declared in 2019 presented at the Third Global Ministerial Summit on Patient Safety [25]. In view of this initiative to promote safety, they have affirmed the need to include patient safety in the undergraduate curriculum for Medical, Nursing, Dental, and Allied Health Sciences (and related) degrees and use innovative styles for the training of health professionals such as Inter-Professional Education. Well-designed educational materials and workshops on patient safety may enhance pharmacy students’ knowledge about patient safety. A systematic review of forty-one curricula has reported that having enough numbers of faculty familiar with patient safety content, setting competing educational needs, and ensuring learners’ enthusiasm are all factors that affected the successful curricular integration [8].

To the best of our knowledge, this is the first study evaluating the attitude of pharmacy students towards patient safety in Saudi Arabia. Our study builds on earlier international studies highlighting the need to integrate patient safety into the pharmacy curriculum [13–17]. It highlights a knowledge gap in patient safety among pharmacy students in Saudi Arabia. This calls for a national thorough review of pharmacy curriculums to assess the gaps and needs. There are some limitations to the findings of the present study. First, the study was conducted in four pharmacy school which may limit the generalizability of the findings. Also, we have not conducted a regression analysis, therefore, we did not control for confounding variables which is a major threat to the validity of inferences. Besides, this was a questionnaire-based study and therefore it is subject to recall bias. In addition to the previously listed limitations, the study design which is a descriptive study and no data was collected about impacts of the educational program in attitude towards patient safety worth to be considered in future research. Besides, no information was collected about the background data for the participants involved in this study. Yet, this study may add to the current data as a base for future research exploring patient safety among healthcare professionals. Further, this study had tried to pinpoint potential factors that may affect attitude towards patient safety and therefore shall help future research to consider these factors and what research gap needs to be filled.

## Conclusions

Pharmacy students from different levels and colleges have shown a positive attitude toward learning about different topics in patient safety. Hence, such findings should inform policymakers in the educational and health sectors in the Kingdom about the need to incorporate patient safety courses in all pharmacy programs given its impact on patient care.

## Data Availability

The dataset supporting the conclusions of this article is available by request from the corresponding author.

## Abbreviations

APSQ-III: Attitudes to patient safety questionnaire III
WHO: World health organization
